# Calculation of the Probability of Survival for Trauma Patients Based on Trauma Score and the Injury Severity Score Model in Fatemi Hospital in Ardabil

**DOI:** 10.5812/atr.9411

**Published:** 2013-06-01

**Authors:** Vadood Norouzi, Iraj Feizi, Soodabe Vatankhah, Majid Pourshaikhian

**Affiliations:** 1 Department of Anesthesia, Ardabil University of Medical Sciences, Ardabil, IR Iran; 2 Department of Surgery, Ardabil University of Medical Sciences, Ardabil, IR Iran; 3 Department of Health Care Services Management, Tehran University of Medical Sciences, Tehran, IR Iran

**Keywords:** Wounds and Injuries, Survival, Mortality

## Abstract

**Background:**

Trauma, in addition to economic and social costs, is the fourth cause of death in the world and in the year 2000 alone, it led to the death of more than 6000000 people. In Iran, Trauma has the first burden of disease and also needs a long medical surveillance.

**Objectives:**

The aim of this study was to evaluate the outcome of trauma cases using the trauma score and the injury severity score (TRISS) model and then comparing this with the results of a major trauma outcome study (MTOS) carried out in the US.

**Patients and Methods:**

This study is a retrospective, descriptive and analytical study on 1000 patients aged 2 - 82 years old with closed or penetrating traumas staying at Ardebil Fatemi hospital. In this study, injury severity score (ISS), revised trauma score (RTS), and TRISS were calculated and patients' viability ratios were obtained.

**Results:**

The results showed that 714 patients (71.4%) were male and 286 patients (28.6%) female with the mean age of 35.68 years. In this study 45 (4.5%) and 955 patients (95.5%) had penetrating and blunt traumas, respectively, whereby the head and neck were the most prevalent (74%) areas of injury. The most common reason for these traumas was, accident with vehicles with 670 cases (67%), which resulted in hospitalization. From this group, ninety-seven cases (9.7%) died in the hospital. From these results, calculations of ISS and RTS were 15.50 ± 11.31 and 7.49 ± 0.79, respectively. According to the calculation of the TRISS model, 91.5% of trauma victims should be survived, while only 90.3% survived practically.

**Conclusions:**

We can conclude that the surveillance presented to our injured group probably had some defects that need to be revised in therapeutic services to enhance survival requirements.

## 1. Background

A trauma is any injury or damage that occurs due to external factors. Traumas resulted in the death of more than 6000000 people in the world, during the year 2000. Trauma is one of the biggest causes of disability in young people and also it is the single greatest cause of years of life lost in the world. Trauma, in addition to socio-economic costs on the society and long-term medical needs, is the fourth leading cause of death in the world. In developing countries, trauma is the first cause of death in young people. Road traffic accidents, after the cardiovascular diseases, is known as the second leading cause of death in Iran. (1, 2). In recent years, efforts have been made to standardize the treatment of trauma patients, yet, 50% of deaths occur in these patients at the field. These patients are classified as first stage. In the second stage, trauma centers may have the greatest involvement and 30% of trauma deaths are in this category. It has been shown that the involvement of trauma systems and treatment centers, decrease mortality from 30% to 9%. The third stage is between 1 and 30 days after the trauma and about 10 - 20% of deaths occur during this period (3, 4). Patient assessment has a vital role; this includes an initial assessment of the injury (assessment of airway, respiration and circulation) and a secondary assessment based on a systematic method (1-5). The scoring method of the trauma consists of four components which assess prevention of damage, prediction of injury severity, mortality and improvement in the quality of hospital services (3-5). The qualitative scoring is the only available standard for epidemiological studies and a source of comparison of treatment methods between different individuals (6, 7). For this purpose, the TRISS model has been widely used and includes three types of strategies; physiological, anatomical and the combination of the two. The revised trauma score (RTS) is a physiologic evaluation criterion for predicting in-hospital mortality and the outcome of the patient's trauma and has five independent variables, including glasgow coma scale (GCS), respiratory rate (RR), systolic blood pressure (SBP), chest expansion and capillary refill. The ranges of RTS are from 0 to 12 and the lowest grade represents the most severe trauma. In the RTS, GCS has the highest coefficient of credibility and influence in the prognosis of patients with head injury (1-3, 8). The criteria for measuring the trauma severity (ISS) is determined based on the anatomical location of the lesion. The combination of the trauma score and the injury severity score (TRISS) is used to evaluate treatment and is a strong indicator of the expected mortality in trauma patients while age is considered as a factor in patient's survival. This calculation is based on a major trauma outcome study (MTOS) from the College of Surgeons of America. Also, this model is used to compare different hospitals and health care services (8-11). In the study that was conducted by Khosravi et al. at Imam Hussein hospital in Shahrood which was "the study of trauma patients admitted to the hospital, using TRISS" 47 trauma deaths occurred , but based on the TRISS model only 35 deaths were expected and the probability of survival for all patients were 82.7% (chance of death 17.3%). In this study, it was observed that the number of death was 4 cases per 100 patients that were more common than MTOS study. The MTOS study was shown that the quantitative assessment of services can be a suitable criteria for comparing and evaluation of health services before and after admission in trauma patients (12).

## 2. Objectives

In the present study, as a result of numerous types of trauma patients, who have been admitted to Fatemi hospital, in Ardabil, after a review of patients based on the TRISS score (6-8), they were monitored for survival, and mortality.

## 3. Patients and Methods

The present study is a retrospective, descriptive and analytic study that was conducted on 1000 patients with multiple traumas, aged between 2 - 82 years in Ardabil Fatemi hospital, in 2011. Inclusion criteria included the existence of multiple traumas caused by road accidents and other events and exclusion criteria was trauma only in one area of the body. The causes of trauma were divided into four categories: 1) trauma due to vehicle accidents, 2) injury caused by downfall from a height, 3) trauma caused by assult and 4) trauma caused by motor vehicle pedestrian accidents. Data were collected using a TRISS questionnaire and trauma patient’s records and the severity of trauma damages were entered in the TRISS software in order to estimate probability of survival. Data were analyzed using SPSS 16, Chi-square and t-tests, and P < 0.05 was considered significant. The Calculation of ISS criteria based on type and location of injuries according to the classification of abbreviated injury scale (AIS) was measured. In this method, the highest score of each of the six regions of the body (head, face, chest, abdomen, pelvis, limbs and extremities) were used and the three highest scores were squared, and the sum was considered as criteria for measuring of the ISS. The range of ISS scores were between 0 - 75 increasing with the severity of injury. If the damage was severe enough to be irreversible, AIS were awarded and the criteria for measuring the ISS would be equal to 75 (ISS = 75). To calculate the RTS, the three indicators, including the GCS, SBP and RR were measured with each divided into 4 categories and a number between 0 (worst) to 4 (best) was allocated. Codes from Table 1 were multiplied with each index (GCS = 0.9368, SBP = 0.7328 and RR = 0.2908) and the product of the multiplication determined the total number for RTS. The logistic regression used to predict and analyze the outcomes of injury and the probability of survival is calculated based on the standard of RTS (Table 1).

**Table 1.** Method of Calculation of Revised Trauma Score

GCS ^a^	SBP ^a^	RR ^a^	Coded value
**13 - 15**	> 89	10 - 29	4
**9 - 12**	76 - 89	> 29	3
**6 - 8**	50 - 75	6 - 9	2
**4 - 5**	1 - 49	1 - 5	1
**3**	0	0	0

^a^Abbreviations: GCS, glasgow coma scale; SBP, systolic blood pressure; RR, respiratory rate

To estimate the probability of patient survival by TRISS, scores of RTS, ISS and age (based on age groups;< 15 years, 15 - 45 years and above) are inserted in the following formula. For < 15 years and 15 - 45 year groups, the coefficients are equal. In the < 15 year group, survival from the penetrating trauma is calculated as the same as the blunt trauma model: A) Penetrating trauma: Logit = -2.5355 + RTS × 0.9934 + ISS × 0.0651 + (Age points) × 1.1360, B) Blunt trauma: Logit = -0.4499 + RTS × 0.8085 + ISS × 0.0835 + (Age point) × 1.7430. In both cases the probability of survival is equal to equation 1: Probability of survival (Ps) = 1/ (1 + elogit). The coefficients of the TRISS model are based on the results of the MTOS study. The collected data were analyzed using SPSS software and patients’ survival and death was predicted based on the TRISS model. The product of the probability of patients’ mortality and the expected number of deaths were calculated. The coefficients of RTS and ISS and age variables according to the MTOS study - are placed in the equation, as bellow (Table 2):

Equation 2: b = b0 + b1 (RTS) + b2 (ISS) + b3 (age).

Since each of these variables has a different value for each patient, as mentioned in Equation 2, the value of b is obtained from each individual (Table 2). Then this value is placed in equation 1 and the probability of survival of each case was calculated based on the MTOS coefficients. After the calculations, patients who have a survival probability of more than 0.5 will survive and patients with a score of less than 5 must die accordingly. It has been observed that the number of deaths that occurred are similar to that expected (Table 2).

**Table 2.** Coefficients Required for Calculating of the Survival Probability

Trauma	b3 ^a^	b2 ^b^	b1 ^c^	b0
**Blunt**	-1.9052	-0.0768	0.9544	-1.2470
**Penetrating**	-2.6676	-0.1516	1.1430	-0.6029

^a^Age

^b^ISS

^c^RT

## 4. Results

**Figure 1.**
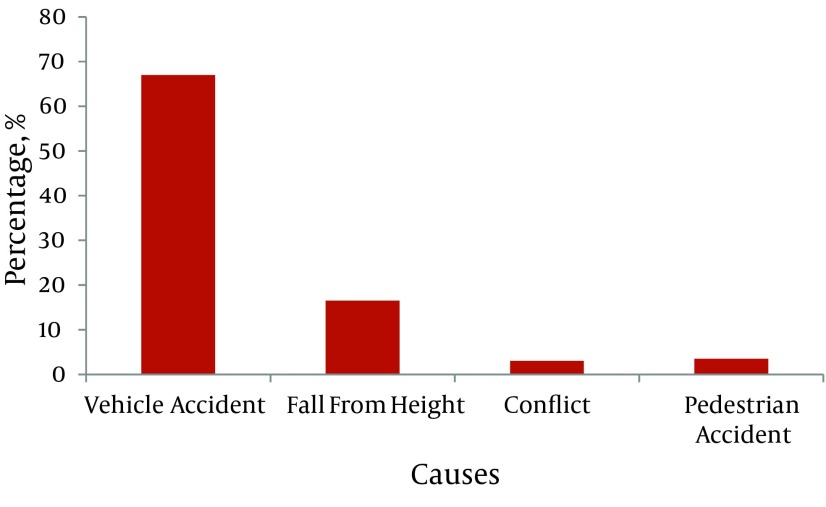
Relative Frequency of Patients Hospitalized Due to Injuries

The results showed that 714 patients (71.4%) were male and 286 (28.6%) female. The mean age of patients was 35.68 ± 20.62 years and the majority of trauma accidents (24%) was in the age group 13 - 22 years. In this study, we observed the damage to the body and found that the most common (75%) sites of injury are the head and neck. The mean duration of hospitalization for patients was 7.4 ± 3.2 days. According to trauma-related injuries, 45 (4.5%) and 955 patients (95.5%) have had penetrating and blunt traumas, respectively. The most common cause of injury was vehicle accidents with 670 cases (67%) (Figure 1).

In this study, trauma causes were classified according to sex. The most common cause of hospitalization due to injury in men and women was trauma from road accidents and there was a significant correlation between types of trauma and gender (P = 0.000). To find the most common cause of trauma in each age group, the causes of trauma in patients of different age groups were evaluated separately (Table 3).

**Table 3.** Frequency of Injuries in Different Age Groups and Trauma Causes

Age Group, y	Pedestrian Accident, No. (%)	Conflict, No. (%)	Dropped, No. (%)	Vehicle Accident, No. (%)	Total
**2-12**	0 (0)	0 (0)	40 (44.4)	50 (55.6)	90
**13 - 22**	45 (18.8)	12 (5)	32 (13.3)	151 (62.9)	240
**23 - 32**	25 (11.1)	12 (5.3)	46 (20.4)	142 (63.1)	225
**33 - 42**	15 (13)	1 (0.9)	17 (14.8)	82 (71.3)	115
**43 - 52**	30 (26.1)	0 (0)	15 (13)	70 (60.9)	115
**53 - 62**	5 (8.3)	0 (0)	0 (0)	55 (91.7)	60
**63 - 72**	5 (6.2)	5 (6.2)	5 (6.2)	65 (81.2)	80
**73 - 82**	10 (13.3)	0 (0)	10 (13.3)	55 (73.3)	75
**Total**	135 (13.5)	30 (30)	165 (16.5)	670 (67)	1000

The results showed that 97 cases (9.7%) died in hospital and the largest percentage of deaths in the age group 23 - 32 years. The results were based on the number of people who died; 10 (22.2%) of which had penetrating traumas, while 87 (9.7%) had blunt traumas. Data analysis showed a significant correlation between mortality and type of injury (P = 0.004). It was observed that the mean of ISS was 15.50 ± 11.31 and mean of dead patients was significantly higher than the living. The mean ISS in patients with penetrating trauma was significantly more than those with blunt trauma. The study found that the average RTS among trauma patients was 7.49 ± 0.79. The RTS average showed a significant correlation between the dead and those who survived (Table 4).

**Table 4.** The ISS and RTS Based on Condition and Type of Trauma

	ISS ^a^ Score	ISS^a^, P value	RT^a^ Score	RT^a^, P value
**Condition of Patient**		0.000		0.000
Dead	14.12± 29.65		5.29 ± 1.35	
Alive	9.83 ± 13.98		7.62 ± 0.57	
**Type of Trauma**		0.034		
Penetrating	12.51 ± 19			
Blunt	11.23 ± 15.34			

^a^Abbreviations: ISS, injury severity score; RTS, revised trauma score

In this study, the probability of mortality- according to MTOS study- was 8.5%, thus, the expected number of death and survival patients was 85 and 91.5, respectively. The W statistic was equal to -3 and Z statistic was + 0.19. Also, according to the TRISS model on a thousand patients, the risk of death in 85 cases was more than 50% (expected death), so the standardized mortality ratio was 1.14, i.e. if the patients treated in a standards trauma center (based on MOTS), the mortality rate of patients was lower, 1.14 deaths per 100 persons. In the next stage, using the coefficients provided by the MTOS study, the probability of survival was calculated for each patient. Based on this calculation in patients with trauma, 91.5% of cases should be alive; while actually 90.3% survived (Table 5).

**Table 5.** Classification of Patients with Trauma and Mortality

	Expected Death		Incidence of Death	
	Penetrating	Blunt	Penetrating	Blunt
**Died**	8	77	10	87
**Lived**	37	878	35	867

## 5. Discussion

In developed countries the TRISS qualitative criteria are used for assessment and calculation of injuries and the survival probability in trauma patients. However, in developing countries, a few studies have been done using this index to evaluate the severity of the trauma (12-14). In this study, results based on the TRISS model, has been compared with normal values obtained from studies conducted in North America titled “major trauma outcome study” (4). In this study, 46.5% of the patients who were admitted to the emergency ward had a lower age compared to previous studies, yet, these results are similar to the results of most previous studies (15). The results of this study showed that the majority of trauma patients were men similar to studies conducted in other countries, as men are more prone to accidents and crashes than women (13, 14, 16). Traffic accidents were the major cause of trauma and this was consistent with the results of Zafar (17) and Solagberu (18) studies. Head and neck injuries were the most common injuries compared to other parts of the body. The study that was conducted by Dr. Zafar in Pakistan, showed that among the 14 cases of death due to trauma, 13 cases were caused by injury in the head and neck (14). In the present study, the average RTS score in living patients was 7.62 ± 0.57, and 5.29 ± 1.35 in dead cases. This finding has no significant difference with Zafar (17) and Murlidhar in India (19) that reported the mean of 7.57 and 4.9 for survival and deaths, respectively, and a significant difference with the results of study of Khosravi were observed (20). In this study, the results showed that the ISS is also significant in patients who died as well as those who survived. In a study conducted by Champion and colleagues (15) performed on 3833 patients, mortality and complications of disease significantly increased with increasing severity of the ISS index. The correlation between ISS score and mortality of trauma patients has been studied by several researches and the results of all studies have suggested a relationship between the severity of injury and death in trauma patients (11, 21-23). In the present study, we found that an increase in ISS score above 25 is directly associated with increased risk of death in injured patients. The coefficient that we obtained in our logistic model was different from the coefficient that was provided by the study of MOTS. Since there is a variable component in the ISS in patients with penetrating traumas as well as the impact of age on survival, it can be concluded that the patients in our study group in terms of outcome, were worse than MOTS study. Z statistic records the difference between the observed and expected numbers of deaths. In our study, the group with blunt traumas had Z = 6.14 and in penetrating trauma Z was 3.4 where both cases were significant. The difference in the outcome between the two groups can be because the injured patients had a more severe injury or care provided was not enough. For the first condition, we used the M statistic that compares the ratio of probability survival (Ps) of patients in different degrees. The M statistic in our study was 0.91, which means the low similarity between victims of our study and MTOS study. We understood that our victims have had more severe traumas. Therefore, we can conclude that the provided care to the patients have probably had shortcomings. Of course, this may be due to trauma systems in western countries where they provide appropriate care to patients at the scene of the accident by trained staff. According to the results of the present study, using quantitative criteria of TRISS for evaluating the quality of health service, according to the above results and using the quantitative criteria of TRISS to evaluate the quality of health care services, it seems that Fatemi hospital provided relatively good care for trauma patients, although there were defects. Therefore, it is necessary to review their medical services in order to increase survival in trauma patients.
